# Tailoring work participation support for cancer survivors using the stages of change: perspectives of (health care) professionals and survivors

**DOI:** 10.1007/s11764-022-01196-x

**Published:** 2022-03-11

**Authors:** Amber D. Zegers, Pieter Coenen, Ute Bültmann, Ragna van Hummel, Allard J. van der Beek, Saskia F. A. Duijts

**Affiliations:** 1 Department of Public and Occupational Health, Amsterdam UMC, Vrije Universiteit Amsterdam, Amsterdam Public Health Research Institute, van der Boechorststraat 7, 1081 BT Amsterdam, The Netherlands; 2 Department of Health Sciences, Community and Occupational Medicine, University of Groningen, University Medical Center Groningen, Groningen, The Netherlands; 3 Re-turn, Cancer-Related Return-To-Work Consultancy and Guidance, Utrecht, The Netherlands; 4 Department of Research & Development, Netherlands Comprehensive Cancer Organization, Utrecht, The Netherlands

**Keywords:** Cancer survivors, Return to work, Work retention, Stages of change

## Abstract

**Purpose:**

Interventions to support work participation in cancer survivors (CSs) have shown limited effectiveness. Applying a behavioral change framework (e.g., stages of change) could make work participation interventions for CSs more timely and tailored. We aimed to explore the application of the stages of change framework to work participation support for CSs and to generate stage-specific intervention content.

**Methods:**

Eighteen experts (e.g., occupational physicians, reintegration consultants) were individually interviewed, and three focus groups with CSs (*n* = 6, *n* = 5, *n* = 4) were conducted. Data were analyzed along the six work-related behavioral change stages purported by the readiness for return to work framework, which is based on the stages of change.

**Results:**

The following themes were identified: (1) pre-contemplation: emotional support and staying connected—encourage contact with the employer/colleagues; (2) contemplation: considering return to work is stressful—facilitate the deliberation process; (3) preparation self-evaluative: assess current capabilities—seek guidance from, e.g., occupational physicians; (4) preparation behavioral: planning return to work—allow for personalized solutions and encourage supervised return to work; (5) uncertain maintenance: guard against overload—train self-efficacy strategies and communication tactics; and (6) proactive maintenance: accept and prepare for the long term.

**Conclusions:**

Our results support the potential utility of tailoring CSs’ work participation support along the stages of change.

**Implications for Cancer Survivors:**

We provided recommendations for intervention content and developed a stage-specific work participation intervention for CSs, the effectiveness of which will be evaluated in an upcoming randomized controlled trial.

**Supplementary Information:**

The online version contains supplementary material available at 10.1007/s11764-022-01196-x.

## Introduction

While cancer incidence continues to rise, improvements in screening, treatment, and survivorship care allow cancer survivors (CSs) to live longer, with better quality of life [1, 2]. Due to these advancements, CSs are often able to look beyond the burden of the disease and its treatment, towards reconstructing daily life. As approximately 40–50% of CSs are of working age at diagnosis [3], many will have to consider working with, or after, cancer. From a personal perspective, being able to work largely contributes to CSs’ rehabilitation and supports a sense of normalcy, identity, purpose, and financial independence [4–6]. From a societal perspective, the financial burden of cancer due to, for instance, productivity loss [7] necessitates that CSs who are able and wish to participate in work should be supported to do so. However, return to work (RTW) is a complex and multifaceted process that is influenced by, among others, disease and treatment-related, personal, occupational, and legislative factors [8–10]. Hence, CSs are 1.37 times more likely to be unemployed compared to healthy (non-cancer) control participants [11].

Various (health care) professionals are involved in the RTW process, e.g., medical specialists, occupational therapists, physiotherapists, general practitioners, occupational physicians, insurance physicians, and supervisors/employers. However, central to the RTW process are CSs themselves, who often wish to receive guidance throughout this complex trajectory [12, 13]. Although it is known which factors hinder and facilitate RTW in CSs [14–16], interventions to support RTW have not been proven sufficiently effective to be implemented on a wider scale [17, 18]. In a Cochrane review, de Boer and colleagues showed that there was no support for the effectiveness of single-component interventions (e.g., psychoeducational, exercise, or medical interventions), and only weak support for the effectiveness of multi-component interventions (i.e., interventions combining psychoeducational, exercise, and/or vocational components), to improve RTW in CSs [17]. A more recent systematic review showed that interventions to support RTW in CSs did not improve RTW compared to usual care [18]. In addition, interventions to support work retention after initial RTW are scarce.

Recently, researchers have started to apply concepts from behavioral psychology to the RTW/work retention process of CSs. Duijts and colleagues [19] suggested the application of the TransTheoretical Model of health behavior change (TTM) [20], and its core component, i.e., the stages of change, to the development of interventions to support CSs’ RTW. The readiness for return to work (RRTW) scale [21] measures the TTM’s stages of change in the context of work resumption and work retention. Originally developed for lost-time claimants^1^ with musculoskeletal disorders, the RRTW scale has been translated into Dutch and adapted for CSs [22]. The adapted scale indexes information related to six behavioral change stages that CSs may go through in the RTW and work retention process: pre-contemplation (not thinking about work resumption), contemplation (starting to think about work resumption), preparation self-evaluative (starting to conceptualize and prepare for RTW), preparation behavioral (planning RTW and actually going back to work), uncertain maintenance (being back at work but feeling uncertain about being able to retain work), and proactive maintenance (being back at work and feeling able to retain work) [21]. Similar to the TTM [20], the theory behind the RRTW scale [21, 23] purports that individuals move through the stages of change non-linearly (i.e., they can skip stages and lapse back to prior stages).

By identifying CSs’ behavioral change stages related to RTW, interventions to support work participation are assumed to be better able to tap into CSs’ stage-specific circumstances and support needs [18, 19]. In the current study, we qualitatively explored the application of the stages of change [20], using RRTW stage terminology [21, 22] (hereafter “behavioral change stages”), to work participation support for CSs. We conducted this exploration through interviews with various (health care) professionals (hereafter: experts) and focus groups with CSs. Our aims were to (1) explore whether experts and CSs recognized the behavioral change stages in their experiences regarding RTW and work retention, (2) identify CSs’ stage-specific needs and common stage-specific barriers and facilitators for RTW/work retention, and (3) generate stage-specific intervention content to support work participation in CSs.

## Methods

### Design

The current qualitative study is part of the STEPS project (SusTained Employability of cancer Patients and their partnerS), i.e., a combined randomized controlled trial (RCT) for CSs and cohort study for partners of CSs. Within the STEPS RCT, we will evaluate a behavioral change-based intervention program to support RTW and work retention in CSs [24]. The current study was designed and reported based on the Consolidated Criteria for Reporting Qualitative research (COREQ) checklist [25] (Supplementary File 1).

### Sample and recruitment

Experts in the field of cancer and work (e.g., occupational physicians, occupational therapists, reintegration consultants) were eligible to participate in an individual interview if they had experience with work participation support for CSs. Experts were recruited via the professional network of the research team (i.e., purposive sampling) and invited for participation via e-mail or telephone. Hence, a professional relationship between most authors and experts existed prior to study commencement. Upon response to the invitation, experts received an information letter, informed consent form, and a brief questionnaire indexing sociodemographic information and relevant work experience. After signing the informed consent form and completing the questionnaire, an interview was scheduled to take place at the expert’s workplace, at Amsterdam UMC, or via telephone.

CSs were eligible to participate in a focus group if they were between 18 and 65 years of age at the time of inclusion, had been diagnosed with cancer a maximum of 2 years ago, had received chemotherapy or radiotherapy, were currently employed, and had been on sick leave at least some time during the past 2 years. Convenience sampling was applied, as CSs were recruited via various (social media) platforms, including those of the Dutch Federation of Cancer Patient Organizations (Dutch acronym: NFK), and patient panels of “kanker.nl” and the Antoni van Leeuwenhoek hospital. Using a recruitment flyer, CSs were invited to contact the research team, whereupon they received the digital information letter and a link to a digital informed consent form and screening questionnaire, indexing sociodemographic and disease-related information. Eligible CSs were contacted by telephone to schedule a focus group at Amsterdam UMC. Due to this recruitment strategy, no information on non-response is available. No relationship was established with CSs prior to study commencement, excepting two: one participant who was a family member of author PC and one participant who was known to the research team due to her participation in previous psychosocial oncology research projects conducted by the team.

### Data collection

Expert interviews were held between August and November 2019 and had a mean duration of 68 min per interview. Interviews were primarily led by one author (A.Z. or S.D., a PhD candidate and a senior researcher respectively, both with a research focus on psychosocial oncology), with one or two secondary interviewers present to supplement and take notes (A.Z., S.D., or P.C., the latter being a senior researcher with a research focus on occupational epidemiology). A.Z., S.D., and P.C. have experience in conducting qualitative research [22, 26]. Interviews were semi-structured and followed an interview guide, which was constructed based on suggestions of prior research [11, 17, 18, 27–31] and was organized along the behavioral change stages [21, 22] (Supplementary File 2). Prior to the interview, experts received the interview guide, which included brief vignettes on the behavioral change stages and suggestions for intervention content. Experts were invited to provide their reactions, alterations, additions, and suggestions in text boxes throughout the document, to be discussed during the interview. The interview guide was not pilot tested. Expert interviews were audio recorded and the data were used to adapt suggested intervention content to be discussed during the focus groups with CSs. A research assistant was present at all interviews to transcribe the main points discussed.

Focus group interviews were held between November 2019 and March 2020 and had a mean duration of 114 min per interview. Focus groups consisted of a maximum of six participants per group and were led by an experienced moderator (SD). Two observers were present during each focus group (A.Z., P.C.), to take notes on non-verbal behavior and ask additional questions. A research assistant was present at all focus groups to observe and take notes. Focus groups were semi-structured and followed an interview guide (Supplementary File 2), which was constructed based on findings of prior research and expert interview data and was organized along the behavioral change stages [21, 22]. Focus group participants received a worksheet detailing the behavioral change stages and suggestions for intervention content, during the focus group. The focus group interview guide was not pilot tested. Focus groups with CSs were audio recorded and transcribed full verbatim by either one author (A.Z.) or a research assistant. To protect the confidentiality, identifiable information was removed from expert interview and focus group transcripts. No repeat interviews or focus groups were held. Transcripts were not returned to participants.

### Analysis

Expert interviews and focus groups with CSs were held until data saturation was reached (i.e., similar (sub)themes were identified from the data in the absence of new information). Expert interview data were not analyzed thematically as expert interviews were held to inform the focus groups with CSs. Main points from expert interviews were collated per behavioral change stage and were presented as a preamble to stage-specific focus group results. Grounded in realist epistemology (i.e., the notion that participants’ perspective and description of the world is identical to reality as they perceive it [32]), focus group data were analyzed using a semantic approach, i.e., themes were identified within the explicit meanings of the data. Thematic analysis was then performed according to the six-step framework of Braun and Clark [33] (i.e., familiarizing oneself with the data, generating initial codes, searching for themes, reviewing themes, defining and naming themes, and producing the report), using ATLAS.ti 8 for Windows (ATLAS.ti Scientific Software Development GmbH). Main themes were based on the theoretical framework underlying the suggested intervention content, i.e., the six behavioral change stages used to structure the expert interviews and focus groups with CSs [21, 22], whereas subthemes were derived from the data.

To assess inter-coder reliability, one focus group transcript was independently coded by two authors (A.Z., P.C.). Afterwards, codes were discussed to reach consensus and to build the final coding tree (Supplementary File 3). A.Z. coded the remaining focus group transcripts, using this coding tree. Any coding ambiguities or additional codes were discussed with a second author (P.C.). Themes were identified from the codes by A.Z. and discussed in a consensus meeting (with P.C. and S.D.). Participants’ quotations were selected from the focus group data to support findings. Quotations were translated to English by the research team. As expert interviews were not transcribed verbatim, expert quotations are not presented in the results. Participants did not provide feedback on the findings of the current study. Demographic questionnaire data were analyzed descriptively using IBM SPSS Statistics version 26.0 for Windows.

## Results

### Sample characteristics

Eighteen experts participated in the interviews (73% female). Three experts did not complete the brief questionnaire due to lack of time resulting from their heavy workload. Thus, excluding the three missing cases from the descriptive analysis, the mean age was 49 years (SD 11), and the mean years of experience with work-related guidance of CSs were 15 years (SD 10). Experts had a wide variety of, and sometimes multiple, occupations. Primary occupations were occupational physician, some of which specialized in oncology (*n* = 4), reintegration coach specialized in oncology (*n* = 3), psychologist specialized in oncology (*n* = 1), rehabilitation physician (*n* = 1), oncological surgeon (*n* = 1), occupational therapist specialized in oncology (*n* = 1), cancer patient advocate (*n* = 1), national policy advisor (*n* = 1), scientific researcher (*n* = 2), and AYA^2^ representative (*n* = 3).

Fifteen CSs (87% female) with a mean age of 47 years (SD 9; range 34–59 years) participated in the focus groups (Table 1). Participants were diagnosed with various cancer types (i.e., breast, ovarian, colon, rectal, testicular, or head/neck cancer), of which breast cancer was most prevalent (53%). Participants received their diagnosis on average 12 months ago (at the time of the interview, SD 6 months). At time of diagnosis, CSs had a part-time (*n* = 9, 60%) or full-time (*n* = 6, 40%) employment contract, while at time of the focus groups, most participants were on sick leave (*n* = 12, 80%). Although information on job type was not collected via questionnaires, focus group data showed that most participants had white or pink-collar jobs.

**Table 1:** Characteristics of cancer survivors (*N* = 15)

	Mean (SD)
Age (in years)	47 (9)
Gender	*n (%)*
Male	2 (13)
Female	13 (87)
Marital status	
Unmarried	5 (33)
Married	5 (33)
Married and living with children	4 (27)
Widowed	1 (7)
Education	
High (university)	4 (27)
Moderate (higher professional education)	11 (73)
Low	-
Primary cancer diagnosis	
Breast^b^	8 (53)
Ovarian	3 (20)
Colon	1 (7)
Rectal	1 (7)
Testicular	1 (7)
Head/neck	1 (7)
Metastasis	
Yes	9 (60)
No	6 (40)
Treatment^a^	
Surgery	14
Radiotherapy	10
Chemotherapy	12
Anti-hormonal therapy	3
Immunotherapy	4
Other	3
Currently cancer free	
Yes	11 (73)
No	4 (27)
Employment status pre-diagnosis^a^	
Full-time contract	6
Part-time contract	9
Entrepreneur	1
Student	1
Current employment status^a^	
Full-time contract	2
Part-time contract	3
Sickness absence at employer	12
Homemaker or informal caregiver	1
Other	1
Breadwinner status	
Yes	9 (60)
No	6 (40)

^a^Outcome categories were not mutually exclusive.

^b^One CS was diagnosed with breast cancer and squamous cell carcinoma.

## Perceived utility of behavioral change stages

Experts and CSs were able to relate their experiences to, and organize their experience along, the behavioral change stages.

You see very clearly that this [RTW/work retention] process is made up of stages, and all of us here are in different stages. [female patient, aged 38 years].

Both experts and CSs talked about the potential utility of having a stage overview, as it could validate and normalize CSs’ stage-specific experiences and provide them with a future perspective.

I would’ve been happy with a program like this [points at the worksheet]. I’m already happy that I’ve got this [stage overview] on paper; I’ll be able to look at it from time to time. [female patient, aged 59 years].

[In response to above] Each time you’re in a stage, you feel like you can’t look beyond that stage. I still get that feeling now [that I’m back at work]. [female patient, aged 52 years].

## Pre-contemplation: emotional support and staying connected

Experts stated that CSs often appeared to be in Pre-contemplation when they were still undergoing treatment. As for stage-specific intervention strategies and tools, experts advised to focus on the emotional needs of CSs and to gently raise awareness of the RTW trajectory and related laws and regulations. Experts suggested that the first professional point of contact in this stage could be e.g., an oncology nurse or a clinical occupational physician.^3^ Experts further recommended encouraging CSs to initiate and maintain contact with their occupational physician, direct supervisor, and colleagues, and to elicit CSs’ natural curiosity about their work. If CSs stay in pre-contemplation long or show particular reluctance to RTW, experts suggested to discuss pre-existent work-related problems, explore CSs’ own expectations regarding their RTW, and explore whether the CS’s social network (e.g., partner or family members) inhibits RTW. According to experts, members of the CS’s social network can express concern about, and disapproval of, RTW.

CSs emphasized that in Pre-contemplation, the physical and mental burden of cancer treatment precluded any thoughts about work. CSs stated that the treatment trajectory and its related effects were often unclear and unpredictable, preventing them from thinking beyond the present moment.

I think it really depends on your circumstances in this stage. I was in this stage when I was literally sick to death of chemo. […] I think if someone initiated a conversation about work, the only thing I could’ve done was cry. [female patient, aged 47 years].

According to CSs, if work was to be mentioned in this stage, it would have to be done with great delicacy, informally, and without pressure to RTW. CSs reported that as work had faded into the background of cancer and its treatment, informal contact with colleagues and employers was considered helpful for emotional processing and gently re-igniting an interest in work. This informal contact helped CSs to slowly progress to another behavioral change stage. Not being contacted by a supervisor or colleagues was considered hurtful and confusing: CSs expressed that during this time, they needed to feel part of the team, but not be pressured in any way to RTW within a certain timeframe.

They [colleagues] came over for a cup of coffee. […] When you’re still in the [treatment] stage, you’re like: ‘Work is over there. Let it be. This is my world now.’ But that changes when – without talking about work – colleagues come over. Then you start thinking about work and how you had a good time with your colleagues. [male patient, aged 34 years].

Keep in contact a bit. Because at a certain point you’re back at work, and I notice now that because of my hair, people don’t recognize me. And that’s of course because my appearance has changed. But I don’t recognize people anymore either, because so much has changed over time. [female patient, aged 38 years].

In both the pre-contemplation and contemplation stage, a need for early information provision concerning relevant legislation was mentioned by CSs.

I’d suggest discussing legislation about work, because I always felt that the law was breathing down my neck. […] I had to go to the occupational physician and he told me about the law and that I had to go to work now, because I was nearing two years of sickness absence.^4^ So actually, I went to work even though at the time, I wasn’t ready for it at all. [female patient, aged 59 years].

After a year [of sickness absence] I started to look into it [sick leave legislation], because I […] heard stories about reintegration at a different employer^5^ and I thought: ‘Ah! I don’t want that at all! That’s not possible!’ [female patient, aged 57 years].

## Contemplation: considering RTW is stressful

Experts characterized the contemplation stage by feelings of confusion, anxiety, and overwhelm in CSs. According to experts, these feelings could be caused by an internal deliberation process related to RTW, e.g., considerations regarding whether the CS *ought to* RTW, *needs to* RTW, *wants to* RTW, *can* RTW, and *what is needed to* RTW. Experts mentioned that, to facilitate this deliberation process in this stage, various intervention strategies could be applied. First, experts advised using motivational interviewing techniques to elicit CSs’ natural curiosity about RTW, their colleagues and workplace. Furthermore, experts suggested encouraging CSs to talk through their expectations regarding RTW and to address anticipated practical and emotional barriers that might hinder RTW. This would also allow CSs to divide RTW into manageable steps and provide an opportunity to address barriers collaboratively with the intervention provider. Furthermore, experts advised that relevant information should be provided and misconceptions should be gently corrected, e.g., information concerning the independent role of the occupational physician and his/her duty of confidentiality, CSs’ and employers’ legal rights and obligations, and the effects of cancer and its treatment on (long term) work ability. According to experts, this information could be provided by, e.g., a reintegration consultant or a (clinical) occupational physician.^6^

CSs described that thoughts and questions about RTW emerged when they started to physically recover from cancer and its treatment, leading to feelings of confusion, worry, and anxiety.

For me, it [chemotherapy] was four to five months of hell and I didn’t know if I’d get out of it. And how I would get out of it. And what would happen after the last one [round of chemotherapy]. […] When the last one was injected, that was the worst one. But after that, I felt like: ‘Okay, I’m starting to feel better and better, and I can think about the next steps.’ [male patient, aged 39 years].

I thought, you know: ‘I’m done [with treatment] now. Everything’s done and I need to be well again.’ But you’re not [well], at all. You don’t know what you’re capable of at all, really. Because not only does your work continue, but the rest [of your life] continues too. […] I panicked. I thought ‘How?! How am I ever going to be able to function at work for a whole day again?’ [female patient, aged 46 years].

If important questions regarding RTW remained unanswered, CSs described that they could potentially stay in contemplation for a long time. According to CSs, guidance from (health care) professionals would help them in making informed decisions about RTW, and progressing to another behavioral change stage.

I think the occupational therapist can – in this Contemplation stage – help you weigh the pros and cons [of RTW]. [female patient, aged 50 years].

It’d be good if someone said that to you, like ‘No, you’re not going to work. You’re first going to think about these questions. And when you’ve thought about them, and you’ve come up with some of your own answers, then we’re going to talk about that [RTW]. [female patient, aged 52 years].

Not all CSs recognized themselves in the contemplation stage. Some reported to have skipped this stage altogether, which could be due to personality (i.e., being more of a “doer” than a “thinker”) and/or coping strategies (i.e., action-focused coping).

I think I skipped this stage. I’m not much of a contemplator. [female patient, aged 52 years].

In my mind, I was already between stage four [Preparation Behavioral] and five [Uncertain Maintenance]: we’re just going to do it [RTW]. [male patient, aged 39 years].

## Preparation—self-evaluative: assessing current capabilities requires guidance

In Preparation—self-evaluative, experts explained that CSs appeared to have made a favorable decision regarding RTW and to have focused their attention on gaining information on *how* to RTW. Regarding intervention content, experts suggested using worksheets and diary exercises to gain insight into CSs’ current daily schedule, energy levels, and work-related capabilities and to compare those to (proximal and distal) RTW goals. According to experts, RTW goal setting should be done in line with the occupational physician’s advice and input from the direct supervisor where applicable. Work tasks should be discussed with the aim of subdividing these tasks into more manageable, buildable ones that could be prepared for, for instance, occupational therapy. Next, experts mentioned that CSs should be encouraged to build on their current capabilities, instead of focusing on capabilities that might have been lost or (temporarily) diminished due to cancer treatment. To be able to RTW over time, experts advised that CSs’ capabilities, such as physical fitness and emotional resilience, should be expanded progressively at home (e.g., by expanding household tasks, learning to take restorative breaks, engaging in physical therapy or fitness, and/or working on emotional processing of the disease and its impact with, e.g., a psychologist). Other recommended strategies included information provision (e.g., on energy management), verbal encouragement, and emotional validation. Experts emphasized the effort and energy it takes for CSs to (plan) RTW and the feelings of frustration and grief that this can bring about.

In preparation—self-evaluative, CSs described seeking information to evaluate whether they were able to RTW and what RTW would look like (e.g., in terms of working hours and work tasks). This evaluative process was often hindered by a lack of input from (health care) professionals regarding the work-related impact of cancer treatment, confusion about where to find guidance, and fluctuating energy levels.

Am I still able to [work] – am I still the same? Do I still have the same amount of energy to do that in the same way as before? It’s quite nerve-wracking to do it [work] again. Like: ‘Can I still do it?’ And, of course, I still can […] But can I still do it in terms of energy? Can I gather the courage? Can I still deal with all of that input? […] I’ll have to experience that myself. No one can predict this for me. [female patient, aged 38 years].

CSs discussed the importance, in this stage, of gaining insight into their current activities, energy levels, and moments of rest, and to adjust their habits and schedule slowly, at home, to make space for future RTW.

Because work does fit [in your daily/weekly schedule], but then the rest [of daily life] doesn’t fit anymore. [female patient, aged 46 years].

Work on that very consciously: your energy levels. Not at work, but just at home. Like, what are you doing? Take a moment of rest. Then do something again. […] You have some instability, at least I do. Like on one day it’s okay, and then the other day it’s crap. […] That’s hard too. [female patient, aged 57 years].

Guidance from supervisors, occupational physicians, and occupational therapists was thought to be helpful in gaining insight into the abovementioned aspects and in planning future RTW:

Unravel the tasks that make up your job. Which elements are in that? [female patient, aged 57 years].

[In response] And also mirror that to where you’re at right now. […] If I can’t read at home, it’s realistic to assume that I can’t read at work either. […] But I am able to do other things. So […] you have to dissect and filter: I can do this, but I can’t do that yet. […] I think you mainly have to look at the possibilities, if you even get this far. Otherwise, you get completely discouraged. [female patient, aged 47 years].

With your supervisor, look at what’s possible regarding the tasks that you are able to do. […] What you’re aiming to take on [in terms of tasks]: are you able to do that? For that, I think you definitely need an occupational therapist. Because he can give you a bit more insight into your [energy] balance. [female patient, aged 57 years].

## Preparation—behavioral: RTW planning should be concrete, flexible, and personalized

According to experts, CSs in preparation—behavioral sought practical guidance to plan and try out RTW. Experts noted that CSs had a tendency to aim for RTW too quickly in this stage (e.g., due to feelings of responsibility towards the employer, or guilt towards colleagues), which was identified as a risk factor for sickness absence recurrence. Regarding intervention content, experts suggested collaboratively developing a concrete, yet flexible, RTW plan in this stage. This RTW plan should consider the needs and suggestions of the CSs, and, if applicable, the occupational physician’s and supervisor’s input. According to experts, CSs should be encouraged to plan their RTW in detail: how to prepare for their first day/week back at work, how to respond to colleagues, how (often) to communicate with their supervisor, which tasks to take up, for how many hours per week, and so on. Experts warned that trying out the RTW plan might lead to CSs encountering obstacles and setbacks. Therefore, experts suggested that RTW planning should be flexible that employers should be made aware of the necessity of this flexibility, that CSs need to be coached throughout the obstacles and setbacks, and that CSs’ successes should be celebrated. Finally, experts proposed that CSs get help or support to build their work-related self-efficacy, by learning and practicing various skills (e.g., time management, self-discipline, cognitive aids, or communication strategies) that could be taught by, e.g., occupational therapists.

CSs described preparation—behavioral as a turbulent and energy-consuming stage in which they often RTW without the appropriate guidance. CSs described that employers and colleagues often had a well-intended “anything goes” attitude towards their RTW: CSs were welcome to determine their own working hours and work tasks. Although this was experienced as welcome freedom when initially returning to work, CSs mentioned that the continuance of this attitude led to stagnation in their RTW process, confusion about expectations, and frustrations due to the performance of menial tasks.

Even though I had a very good relationship with my direct supervisor, well [he said]: ‘Look around a bit, have some coffee.’ Um, yeah, after a month of having coffee and looking around I thought ‘And now?’ [female patient, aged 57 years].

I told him [occupational physician] that I thought it was time for me to get back to work, or that I thought it’d be good for me. But for months now, we’ve been looking for things that I’d be able to do. […] I think he [supervisor] keeps looking for tasks that are similar to the work that I did before. And I’m thinking it’d be nice if that’s the end goal, but the first steps can be different. […] I just notice it’s so hard to explain how complex it actually is. [female patient, aged 47 years].

I didn’t want any stupid little tasks. No. I did everything I could to get back to work and then to come back and do menial tasks like copying? [Indignant] Well, yeah, no. Absolutely not. […] I need a challenge to recover. [female patient, aged 41 years].

CSs expressed the need for concrete and personalized RTW planning. CSs experienced that (health care) professionals involved in the RTW process often assumed to know what was best for them. CSs’ own perceptions of the situation, however, could differ greatly.

He [occupational health expert7] said to me: ‘Maybe it’s too ambitious to go back to work now, but apply for volunteer work, have coffee with the elderly for one or two hours a week.’ With all due respect – because there’s nothing wrong with that – but we [supervisor and CS] walked out of there and my supervisor said: ‘I saw your face in there: we’re not going to do that. We’ll find another solution, because this way you’re actually taking another step back.’ [female patient, aged 47 years].

You notice it [work-related support] is very dependent on the person. I was also offered support from an occupational psychologist because I wanted to go [back to work] too fast. But I also think there are plenty of people who maybe need that little push. [female patient, aged 57 years].

The question is the same for everyone: ‘How do I develop a realistic [RTW] plan?’ That’s similar. The answer to that is just different for everyone. [female patient, aged 49 years].

## Uncertain maintenance: taking it slow—guarding against overload

Experts noted that CSs had a tendency of wanting to do too much, too quickly when they were recently back at work, e.g., working too many hours at once and taking on too many tasks. According to experts, this can lead to CSs getting frustrated, burnt-out, and relapsing into sickness absence. When CSs were recently back at work, experts explained that both CSs and their employers could struggle, because (1) a structured RTW plan might have been missing, (2) the plan needed continuous assessment and adjustment, and (3) the instability of the CS required open and strategic communication. Experts said that the “anything goes” approach mentioned in the previous stage is noticeable in uncertain maintenance too, as employers might be careful not to exert any pressure on the CS. In uncertain maintenance, experts recommended various intervention strategies to prevent sickness absence relapse: first, as in the previous stage: acknowledging and validating CSs’ efforts in RTW and being back at work and, next, allowing CSs to trial their RTW plan and assess and adjust it periodically. Related to this, experts mentioned educating the employer on the importance of their flexibility in the RTW and work retention process, and maintaining awareness of CSs’ energy expenditure and work/life balance, for instance with the help of an occupational therapist or reintegration consultant. Finally, teaching communication strategies to CSs and their employers to communicate expectations, needs, and support during this fluctuating time.

In uncertain maintenance, CSs described a turbulent state of getting to terms with their “new normal” regarding reduced work ability and also wanting to do too much at once. CSs often wanted to prove that they were still a valuable employee that they could still do the work and contribute to the organization and their team. According to CSs, this could result in feelings of insecurity, guilt towards colleagues, work/life imbalances, fatigue, burnout symptoms, and sickness absence relapses.

You want this [being able to work] so much. But of course, you’re limited in what you can do. But you don’t want to admit that, at all, right? […] I really thought like, ‘Yeah, I can’t do anything anymore. I don’t know anything anymore.’ […] And that makes you very insecure. […] I thought I just had to be able to do and know it all immediately again. [female patient, aged 57 years].

I thought I was ready to start doing things again physically, but cognitively I wasn’t feeling quite right yet. I was dealing with a lot of stress. […] The moment I felt a bit better physically, I thought I was on top of the world. […] I just wanted to do something. […] Someone should have said to me: ‘Slow down for now, you have to process some things and deal with that stress’. [male patient, aged 39 years].

During chemotherapy […] I worked on therapeutic basis.8 […] As per January 1st, I’m fully back at work. And it’s harder than I thought. A lot harder. [female patient, aged 56 years].

CSs suggested celebrating small successes to stay motivated during this turbulent time. They often felt they lost sight of how far they had come regarding RTW and needed an external party to remind them of their successes.

Small wins, you know, your confidence builds through that and then you get into a more positive flow. At least that’s what you want. And that takes time. For me, that took a year before I was fully back at work. [female patient, aged 57 years].

[Your supervisor can say to you] ‘I’m so happy you were able to come today, you did well and I’ll see you the day after tomorrow.’ Like that. Clarity. [female patient, aged 41 years].

CSs also suggested utilizing collegial supervision to ensure they maintained their RTW and work retention plan, including limitations set on working hours and work tasks.

My colleagues know that I always work overtime and at one point I said to them ‘Guys, it’s half past one, tell me to go home’. […] And they do. […] Before I relapsed [into sickness absence] I didn’t do that [listen to colleagues]. After I relapsed, […] I do listen to it. [female patient, aged 57 years].

According to CSs, informing colleagues of their current abilities, limitations, instability, and work-related needs was seen as necessary to align mutual expectations. However, CSs also noted that colleagues could feel anxious and uncomfortable around them. CSs suggested that by clearly communicating about (the consequences of) cancer, this discomfort could be alleviated. Both the CS and the employer could play a role in this communication.

They [colleagues] didn’t have all the information and yeah, then I felt a certain resistance [from them] or something, like: ‘She’s there [at work], but actually we can’t count on her [to do the work].’ […] They [colleagues] often feel it’s [cancer] scary as well. [female patient, aged 57 years].

## Proactive maintenance: accept and prepare for the long term

Experts described that CSs in proactive maintenance seemed to have found their foothold at work, though work retention could still be hindered by continued instability, changing energy levels, and long-term consequences and late effects of cancer treatment. For intervention content, experts suggested supporting CSs and their employers by providing information about the long-term consequences and late effects of cancer treatment on work ability (e.g., by utilizing the expertise of reintegration consultants or occupational physicians specialized in oncology), strategizing for when CSs might recognize these consequences and effects (e.g., knowing where to find relevant support), maintaining awareness of energy expenditure and work/life balance (e.g., by utilizing tools provided by an occupational therapist or reintegration consultant), and maintaining periodic work assessments and long-term employer flexibility.

CSs mentioned that they often felt unstable at work for a long time and that they often struggled to maintain a healthy work/life balance. According to CSs, learning to accept cancer-related changes in their lives, including changes in their work ability, characterized the shift from uncertain to proactive maintenance.

With regards to cancer, how long does a process like that take? To feel like you can turn the page, instead of being in the middle of the chapter? I think that takes years. It’s a mourning process that isn’t just finished within a year. And your fear isn’t gone within a year. And your family also deals with cancer. That’ll all last for a while. [female patient, aged 41 years].

It’s only now [being back at work long-term] that you realize: ‘Oh right, I can’t do that anymore. Or, I do things differently. [female patient, aged 49 years].

[In response to above] Yeah, that’s part of the ‘old me’. [female patient, aged 57 years].

Accepting cancer survivorship as part of one’s identity was not important for every CS.

It’s not for everyone. For instance, I’m still aiming to become my old self again. That’s just my goal. [male patient, aged 34 years].

Last, when CSs were back at work long term, they said lasting instability and changeable work ability needed to be evaluated and discussed periodically. According to CSs, aspects that should be discussed during these evaluations included, for instance, working hours, work tasks, energy levels, work/life balance, work satisfaction, and collegial communication.

Periodic evaluations, I think, with the occupational physician. Just to ask how you’re doing along the way. […] You need room to still be instable. And boundaries […] when you want to go too fast, other people will [have to] hit the brakes for you. [female patient, aged 41 years].

[Interviewer in response to above]: How long do you think you’ll need that [kind of support]?

[In response to the interviewer] My whole life, I think. [female patient, aged 41 years].

When you’re back at work a little longer, [let] employers know that you can experience concentration problems and lack of energy in the long term too. [female patient, aged 50 years].

[In response to above] Lack of sleep. [female patient, aged 38 years].

[In response to above] Acknowledge the late effects. [female patient, aged 59 years].

## Discussion

### Main findings

In exploring experts’ and CSs’ perspectives on the application of the behavioral change stages [21, 22] to work participation support for CSs, we found that both experts and CSs were able to recognize and describe their experiences along these stages. Experts and CSs confirmed the non-linearity of stage progression, i.e., they described stage regression and skipping of stages. Finally, stage-specific needs and suggestions for work participation support were amply provided by experts and CSs.

Although RTW and work retention of CSs have long been referred to as a complex process, only recently have researchers started to look deeper into and describe CSs’ progression through this process. It has become clear that this progression is stage specific [10, 18, 34] and looks similar to the stages of change. Furthermore, there are common needs, barriers, and facilitators that characterize the stages. For instance, we learned that in the first stage of the RTW process (i.e., pre-contemplation), CSs used emotion-focused and social coping to endure cancer treatment and recovery. In later stages (i.e., the preparation and maintenance stages), CSs became progressively more open to action-focused, instrumental guidance from, for instance, occupational therapists and reintegration consultants. These results are in line with prior research into the application of the transactional theory of stress and coping to the RTW/work retention process of CSs [10, 34], and with qualitative findings relating to active vs. passive coping strategies of CSs in this process [26]. Brusletto and colleagues have described that, when immediate danger to life is averted and treatment-related side effects ease, CSs tentatively broaden their vision to life with or after cancer. In detaching from the patient role, CSs become open to practical support to reconstruct their daily life, including paid work [34].

Another example concerns the managing of anxiety, overwhelm, and ambiguity towards RTW in the contemplation stage. Prior research has shown the complexity of making decisions regarding RTW [12, 13, 35]. On the one hand, making a weakly informed decision regarding RTW could lead some CSs to RTW too quickly [12], putting them at risk for sickness absence recurrence. On the other hand, CSs could put off the decision to RTW for too long, thereby increasing their risk of financial toxicity [36]. Experts suggested using motivational interviewing techniques to prevent stage stagnation and elicit CSs natural curiosity about their work. Motivational interviewing has often been linked to the TTM [20]. For instance, one could focus on responding to ‘sustain talk’ in earlier behavioral change stages and focus on eliciting ‘mobilizing change talk’ and ‘commitment talk’ in later stages [37]. Motivational interviewing has been successfully used in other behavioral change research among CSs [38].

Although there are common factors characterizing each stage, in the current study, we highlighted the importance of personalized guidance. Within the TTM [20], there are three dimensions of behavioral change that influence and explain the behavioral change stage one is in decisional balance, self-efficacy, and change processes. Franche and colleagues [23] have stated that, in the context of RTW and work retention, these three dimensions are impacted by employee interactions with the workplace, as well as health care and social security systems. In short, the biopsychosocial context of CSs influences their decision to RTW/retain work (decisional balance), the extent to which they feel able to RTW/retain work (self-efficacy), and which actions they engage in or are motivated by to RTW/retain work (change processes). It is therefore crucial to not only consider the behavioral change stage the CS is in, but also the biopsychosocial factors contributing to that stage assignment at that particular moment in the RTW/work retention process.

Two stage-overarching difficulties emerged from our results: timeliness of the intervention and employer involvement. While CSs indicated that they often lacked information on relevant legislation, they also indicated that it might be burdensome to discuss work early on (i.e., just after diagnosis or during treatment), depending on the side effects of cancer treatment. For others, the side effects of cancer treatment are relatively mild and manageable, which can contribute to these CSs feeling more ready to talk about work early on. The importance of discussing work early on in the hospital setting is becoming increasingly recognized [39], especially when considering CSs’ higher risk of becoming unemployed [11] and risks related to financial toxicity [40]. Being able to accurately gauge CSs’ behavioral change stage by using the RRTW scale [22] could provide a framework for when and how to approach a CS about the topic of work.

Moreover, we learned that employers, occupational physicians, and colleagues play a crucial role in various stages of the RTW/work retention process. However, both experts and CSs indicated that supervisors often lack the know-how to guide CSs through this process confidently and appropriately. This is in line with prior research showing the impacts of supervisor support on successful RTW [14, 35, 41, 42], the difficulties that supervisors experience in this process [15], and the development of interventions to support them in this process [43].

## Strengths and limitations

To our knowledge, this is the first study explicitly applying behavioral change theory to the creation of concrete, stage-specific, intervention content for CSs seeking support in the RTW, and work retention process. Results from expert interviews and focus groups with CSs showed high content overlap with each other (i.e., needs, barriers, facilitators, and suggestions for intervention content) per behavioral change stage, further supporting the potential utility of providing tailored, stage-specific support.

Our results should however be viewed in light of the following limitations. First, although data saturation was reached, our results are based on a limited number of interviews with experts and focus groups with CSs. Focus group participant diversity was limited, possibly due to volunteer bias, as most participants were highly educated female breast CSs. Second, expert interviews were not transcribed verbatim, nor were they thematically analyzed based on the same principles as focus group data [33]. Nevertheless, a research assistant transcribed expert interviews as fully as possible during the interviews themselves, based upon which the authors summarized content per behavioral change stage. Expert interview data were used explicitly to gain initial insights and recommendations per stage, based upon which the interview guide for focus groups with CSs was constructed.

Third, as occupational therapists and reintegration consultants have been chosen as intervention providers within the STEPS intervention program, our results may have been biased towards the role of these professionals in the RTW and work retention process of CSs. However, in all interviews and focus groups, the authors asked open-ended questions and encouraged participants to think of any (health care) professional that might be suitable as an intervention provider.

## Implications for research and practice

Conceptualizing work participation support for CSs along the behavioral change stages allows for (1) developing intervention strategies that address predictable stage-specific needs, barriers, and facilitators (Table 2); (2) addressing work early on in a way that CSs are receptive to; and (3) involving relevant parties within the RTW/work retention process when CSs are ready for action-focused support (e.g., the supervisor, occupational physician, insurance physician, and reintegration consultant). Results of the current study underline the importance of involving and educating the employer (e.g., direct supervisor, HR, or occupational physician) on the impacts of cancer and its treatment on workers’ (sustained) employability. An accessible online toolbox for employers could be useful for this purpose [43].

**Table 2:**
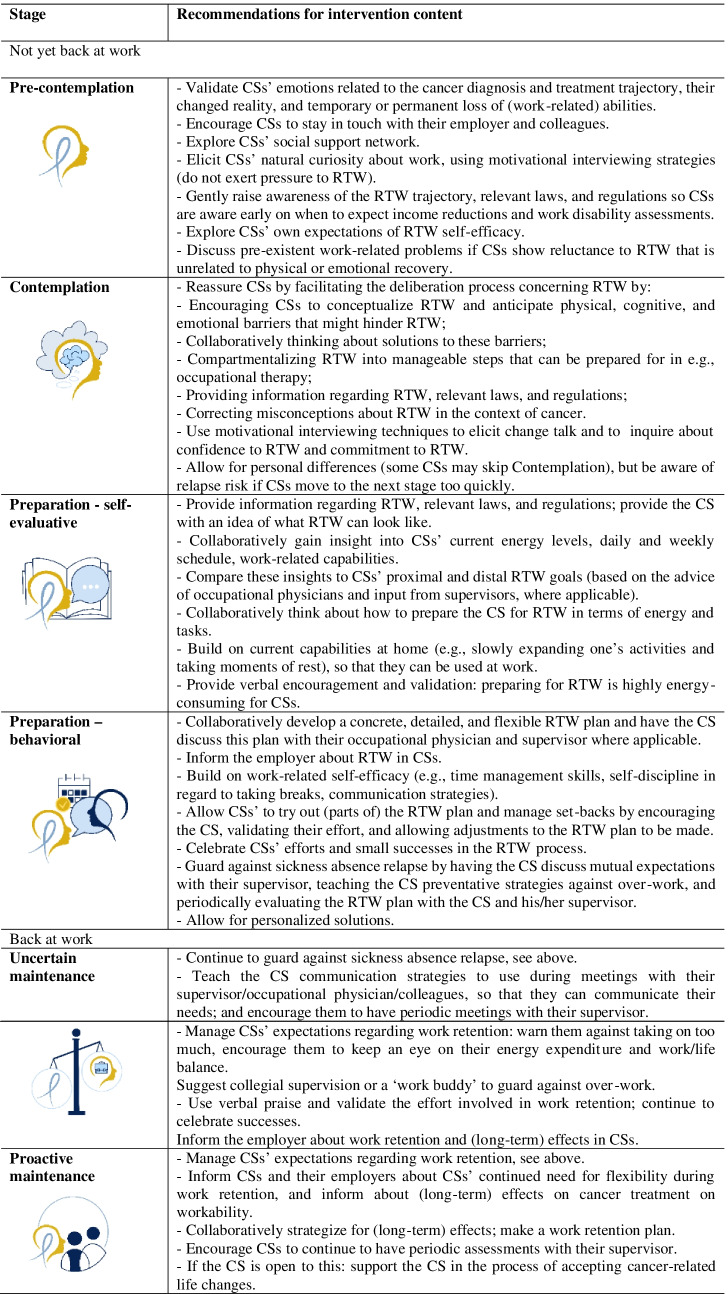
Recommendations for intervention content per behavioral change stage.

Illustrations by Feline Zegers, copyright property of Amsterdam UMC.

The behavioral change stages provide a flexible framework along which to organize RTW and work retention support for CSs and allow room to further tailor intervention content to, e.g., cancer diagnoses, treatment modalities, prognoses, risk factors for unsuccessful RTW, and personal circumstances of CSs. Future research should evaluate the effectiveness of intervention programs that offer stage-specific support for CSs aiming to RTW or retain work. Our research team will do this in an upcoming randomized controlled trial: STEPS [24].

## Conclusion

Our results support the potential utility of organizing work participation support for CSs along the behavioral change stages. From expert interviews and focus groups with CSs, we have distilled stage-specific needs, barriers, and facilitators, which can be used to develop intervention content. Recommendations for such intervention content are presented. Organizing work participation support for CSs in this way provides a clear framework for (health care) professionals involved in the RTW/work retention process of CSs and allows further tailoring based on, e.g., cancer diagnoses, treatment modalities, and personal circumstances of CSs.

## Supplementary Information

Below is the link to the electronic supplementary material.

Supplementary file1 (PDF 171 KB)

Supplementary file2 (PDF 17 KB)

Supplementary file3 (PDF 352 KB)

## Footnotes

1 In Ontario, Canada, a work injury is filed as “lost-time” if, after the day of injury, the worker is unable to go into work, or is able to go into work but is earning less (e.g., because of reduced work hours).

2 This foundation is focused on Adolescents and Young Adults (AYAs) aged 18–39 years, who are diagnosed with cancer.

3 A clinical occupational physician (*klinisch arbeidsgeneeskundige* in Dutch) is a registered occupational physician who has specific knowledge of a certain medical speciality, such as cancer. The clinical occupational physician advises patients, their practitioners, and employers, about questions or problems related to illness and work.

4 According to Dutch legislation, an employee who has been absent from work for 2 years is required to undergo a formal work disability assessment.

5 According to Dutch legislation, the employer and employee are required to assess whether reintegration at the current employer is still attainable. If not, the employer is required to assist the employee in his/her reintegration at a different employer.

6 See footnote 2.
