# Supplementary material for: Tailoring work participation support for cancer survivors using the stages of change: perspectives of (health care) professionals and survivors
Source: J Cancer Surviv. 2022 Mar 11;17(3):706–19. doi: 10.1007/s11764-022-01196-x (PMC10209302; doi:10.1007/s11764-022-01196-x)
Supplement: Supplementary file 1 — Supplementary file1 (PDF 171 KB) [file 11764_2022_1196_MOESM1_ESM.pdf]

Supplementary file 1

Table 1 Consolidated criteria for reporting qualitative studies (COREQ): 32-item checklist

Tailoring work participation support for cancer survivors using the Stages of Change: perspectives of (health care) professionals and survivors

Journal of Cancer Survivorship

Amber D. Zegers[1], Pieter Coenen[1], Ute Bültmann[2], Ragna van Hummel[3], Allard J. van der Beek[1], Saskia F.A. Duijts[1,4]

[1]Department of Public and Occupational Health, Amsterdam UMC, Vrije Universiteit Amsterdam, Amsterdam Public Health Research Institute, Amsterdam, The Netherlands
[2]Department of Health Sciences, Community and Occupational Medicine, University of Groningen, University Medical Center Groningen, Groningen, The Netherlands
[3]Re-turn, cancer-related return-to-work consultancy and guidance, Utrecht, The Netherlands
[4]Department of Research & Development, Netherlands Comprehensive Cancer Organization, Utrecht, The Netherlands

Corresponding author
Pieter Coenen
Department of Public and Occupational Health
Amsterdam UMC, location VUmc
van der Boechorststraat 7
1081 BT Amsterdam, the Netherlands
E-mail: p.coenen@amsterdamumc.nl
Telephone: +31 20 444 8381

No Item	Guide questions/description	Paragraph
**Domain 1: Research team and reflexivity**		
Personal Characteristics		
1. Interviewer/facilitator	Which author/s conducted the interview or focus group?	Heading ‘Data collection’, §1 and §2
2. Credentials	What were the researcher’s credentials? E.g. PhD, MD	Heading ‘Data collection', §1 and §2
3. Occupation	What was their occupation at the time of the study?	Heading ‘Data collection', §1 and §2
4. Gender	Was the researcher male or female?	Heading ‘Data collection', §1 and §2

Supplementary file 1

Table 1 Consolidated criteria for reporting qualitative studies (COREQ): 32-item checklist

5. Experience and training	What experience or training did the researcher have?	Heading ‘Data collection', §1 and §2
Relationship with participants		
6. Relationship established	Was a relationship established prior to study commencement?	Heading 'Sample and recruitment’, §1 and §2
7. Participant knowledge of the interviewer	What did the participants know about the researcher? e.g. personal goals, reasons for doing the research	Heading 'Sample and recruitment’, §2
8. Interviewer characteristics	What characteristics were reported about the interviewer/facilitator? e.g. Bias, assumptions, reasons and interests in the research topic	Heading 'Sample and recruitment’, §2
**Domain 2: study design**		
Theoretical framework		
9. Methodological orientation and Theory	What methodological orientation was stated to underpin the study? e.g. grounded theory, discourse analysis, ethnography, phenomenology, content analysis	Heading ‘Analysis', §1 and §2
Participant selection		
10. Sampling	How were participants selected? e.g. purposive, convenience, consecutive, snowball	Heading ‘Sample and recruitment’, §1 and §2

Supplementary file 1

Table 1 Consolidated criteria for reporting qualitative studies (COREQ): 32-item checklist

11. Method of approach	How were participants approached? e.g. face-to-face, telephone, mail, email	Heading ‘Sample and recruitment’, §1 and §2
12. Sample size	How many participants were in the study?	Heading ‘Results – Demographics’, §1 and §2
13. Non-participation	How many people refused to participate or dropped out? Reasons?	Heading ‘Sample and recruitment’, §2
Setting		
14. Setting of data collection	Where was the data collected? e.g. home, clinic, workplace	Heading ‘Sample and recruitment’, §1 and §2
15. Presence of non-participants	Was anyone else present besides the participants and researchers?	Heading ‘Data collection’, §1 and §2
16. Description of sample	What are the important characteristics of the sample? e.g. demographic data, date	Heading ‘Results – Demographics’, §1 and §2
Data collection		
17. Interview guide	Were questions, prompts, guides provided by the authors? Was it pilot tested?	Heading ‘Data collection’, §1 and §2, Supplementary file 1
18. Repeat interviews	Were repeat interviews carried out? If yes, how many?	Heading ‘Data collection’, §2

Supplementary file 1

Table 1 Consolidated criteria for reporting qualitative studies (COREQ): 32-item checklist

19. Audio/visual recording	Did the research use audio or visual recording to collect the data?	Heading ‘Data collection’, §1
20. Field notes	Were field notes made during and/or after the interview or focus group?	Heading ‘Data collection’, §1 and §2
21. Duration	What was the duration of the interviews or focus group?	Heading ‘Data collection’, §1 and §2
22. Data saturation	Was data saturation discussed?	Heading ‘Analysis’, §1 and §2
23. Transcripts returned	Were transcripts returned to participants for comment and/or correction?	Heading ‘Data collection’, §2
**Domain 3: analysis and findings**		
Data analysis		
24. Number of data coders	How many data coders coded the data?	Heading ‘Analysis’, §3
25. Description of the coding tree	Did authors provide a description of the coding tree?	Heading ‘Data collection’, §3 and Supplementary file 3
26. Derivation of themes	Were themes identified in advance or derived from the data?	Heading ‘Analysis’, §2
27. Software	What software, if applicable, was used to manage the data?	Heading ‘Analysis’, §2 and §3

Supplementary file 1

Table 1 Consolidated criteria for reporting qualitative studies (COREQ): 32-item checklist

28. Participant checking	Did participants provide feedback on the findings?	Heading ‘Analysis’, §3
Reporting		
29. Quotations presented	Were participant quotations presented to illustrate the themes / findings? Was each quotation identified? e.g. participant number	Heading ‘Analysis’, §3
30. Data and findings consistent	Was there consistency between the data presented and the findings?	Supplementary file 3 (coding tree)
31. Clarity of major themes	Were major themes clearly presented in the findings?	Heading ‘Results'
32. Clarity of minor themes	Is there a description of diverse cases or discussion of minor themes?	Heading ‘Results’
