# Supplementary material for: Tailoring work participation support for cancer survivors using the stages of change: perspectives of (health care) professionals and survivors
Source: J Cancer Surviv. 2022 Mar 11;17(3):706–19. doi: 10.1007/s11764-022-01196-x (PMC10209302; doi:10.1007/s11764-022-01196-x)
Supplement: Supplementary file 2 — Supplementary file2 (PDF 17 KB) [file 11764_2022_1196_MOESM2_ESM.pdf]

Supplementary File 2

Table 1 Semi-structured interview guide (expert interviews and focus groups)

Journal of Cancer Survivorship

Amber D. Zegers[1], Pieter Coenen[1], Ute Bültmann[2], Ragna van Hummel[3], Allard J. van der Beek[1], Saskia F.A. Duijts[1,4]

[1]Department of Public and Occupational Health, Amsterdam UMC, Vrije Universiteit Amsterdam, Amsterdam Public Health Research Institute, Amsterdam, The Netherlands
[2]Department of Health Sciences, Community and Occupational Medicine, University of Groningen, University Medical Center Groningen, Groningen, The Netherlands
[3]Re-turn, cancer-related return-to-work consultancy and guidance, Utrecht, The Netherlands
[4]Department of Research & Development, Netherlands Comprehensive Cancer Organization, Utrecht, The Netherlands

Corresponding author
Pieter Coenen
Department of Public and Occupational Health
Amsterdam UMC, location VUmc
van der Boechorststraat 7
1081 BT Amsterdam, the Netherlands
E-mail: p.coenen@amsterdamumc.nl
Telephone: +31 20 444 8381

**Interview topic**	**Expert interview**	**Focus group**
Introduction round	The interviewer introduces herself to the interviewee and asks the interviewee to elaborate on their experiences with providing work participation support for cancer survivors.	The moderator introduces the research team to the interviewees and asks the interviewees to state their first name, cancer diagnosis, the date of diagnosis, and current work status (sick-listed, reintegrating, or fully reintegrated).
Theoretical background and behavioral change stages (Readiness for Return to Work scale, RRTW)	Prior to the interview, the interviewee received a preparatory document. This document followed the content of the interview guide and contained RRTW behavioral change stage vignettes and proposed stage-specific intervention content based on the scientific literature and opinions of the research team. The behavioral change theory and RRTW stages are discussed, and the interviewee is provided with the opportunity to ask questions.	The moderator briefly explains the behavioral change angle taken to work participation of cancer survivors, and explains the RRTW behavioral change stages. Interviewees are given the opportunity to ask questions.
RRTW stage-specific experiences, needs, and intervention content	Per RRTW stage, interviewees are asked whether they recognize the stage in their experiences with providing work participation support for cancer survivors. Stage-specific experiences (needs, barriers, facilitators) are discussed, as well as intervention strategies and tools to support cancer survivors in each stage.<br><br>Example questions:<br>- *Please read the suggested intervention content above. To what extent do you agree with this approach in this stage?*<br>- *In terms of guidance and support, what else do you think is needed in this stage?*<br>- *Which professionals should be involved in this stage and what would be their role?*	Per RRTW stage, interviewees are asked whether they recognize themselves and their experiences with work participation in the stage. They are asked to reflect on their stage-specific needs, and barriers and facilitators for work participation in each stage. Then, interviewees are asked to read and reflect on the proposed stage-specific intervention elements derived from the literature and expert interviews (using a worksheet). Finally, interviewees are asked to provide suggestions for stage-specific support.<br><br>Example questions:<br>- *In this stage, what kinds of obstacles did you encounter with regard to going back to work?*<br>- *What would have helped you in this stage, to progress to another stage?*<br>- *Imagine that someone remains in this stage for a prolonged time, what do you think would be helpful to offer in terms of support?*<br>- *What would have helped you to feel more self-efficacious/confident/secure in this stage?*

Supplementary File 2

Table 1 Semi-structured interview guide (expert interviews and focus groups)

	- *Do you know of any strategies or tools that could be used in this stage?*	- *Looking back on the support that you have received, what was particularly helpful or supportive to you and why?*
Wrap up	The interviewer summarizes the most important conclusions and suggestions provided by the interviewee and thanks the interviewee for participation. The interviewee is given the opportunity to provide additional comments and feedback, and to ask questions.	The moderator summarizes the most important conclusions and suggestions provided by the interviewees and thanks the interviewees for participation. The interviewees are given the opportunity to provide additional comments and feedback, and to ask questions.
