# Supplementary material for: Tailoring work participation support for cancer survivors using the stages of change: perspectives of (health care) professionals and survivors
Source: J Cancer Surviv. 2022 Mar 11;17(3):706–19. doi: 10.1007/s11764-022-01196-x (PMC10209302; doi:10.1007/s11764-022-01196-x)

Supplementary File 3

Figure 1 Coding tree

Journal of Cancer Survivorship

Amber D. Zegers[1], Pieter Coenen[1], Ute Bültmann[2], Ragna van Hummel[3], Allard J. van der Beek[1], Saskia F.A. Duijts[1,4]

[1]Department of Public and Occupational Health, Amsterdam UMC, Vrije Universiteit Amsterdam, Amsterdam Public Health Research Institute, Amsterdam, The Netherlands
[2]Department of Health Sciences, Community and Occupational Medicine, University of Groningen, University Medical Center Groningen, Groningen, The Netherlands
[3]Re-turn, cancer-related return-to-work consultancy and guidance, Utrecht, The Netherlands
[4]Department of Research & Development, Netherlands Comprehensive Cancer Organization, Utrecht, The Netherlands

Corresponding author
Pieter Coenen
Department of Public and Occupational Health
Amsterdam UMC, location VUmc
van der Boechorststraat 7
1081 BT Amsterdam, the Netherlands
E-mail: p.coenen@amsterdamumc.nl
Telephone: +31 20 444 8381

Supplementary File 3
Figure 1 Coding tree

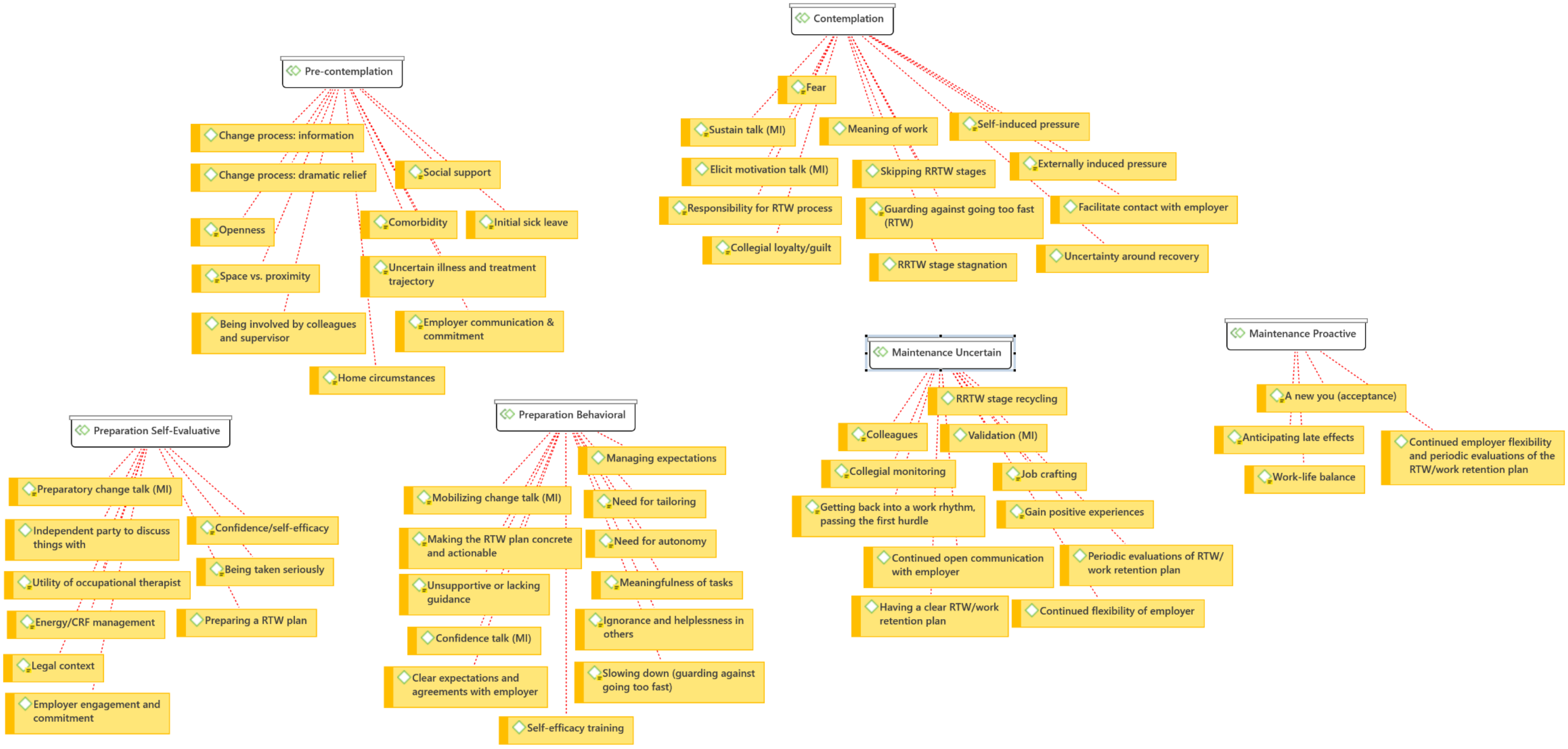

Supplement: Supplementary file 3 — Supplementary file3 (PDF 352 KB) [file 11764_2022_1196_MOESM3_ESM.pdf]
